# Computational and Experimental Drug Repurposing of FDA-Approved Compounds Targeting the Cannabinoid Receptor CB1

**DOI:** 10.3390/ph16121678

**Published:** 2023-12-02

**Authors:** Emanuele Criscuolo, Maria Laura De Sciscio, Angela De Cristofaro, Catalin Nicoara, Mauro Maccarrone, Filomena Fezza

**Affiliations:** 1 Department of Experimental Medicine, Tor Vergata University of Rome, Via Montpellier 1, 00121 Rome, Italy; emanuele.criscuolo@alumni.uniroma2.eu (E.C.); catalin.nicoara@alumni.uniroma2.eu (C.N.); 2 Department of Medicine, Campus Bio-Medico University of Rome, Via Alvaro del Portillo 21, 00128 Rome, Italy; marialaura.desciscio@uniroma1.it (M.L.D.S.); angela.decristofaro@alumni.uniroma2.eu (A.D.C.); 3 Department of Biotechnological and Applied Clinical Sciences, University of L’Aquila, Via Vetoio, Coppito, 67100 L’Aquila, Italy; 4 European Center for Brain Research/Santa Lucia Foundation IRCCS, Via Del Fosso di Fiorano 64, 00143 Rome, Italy

**Keywords:** cannabinoid receptor 1, drug repurposing, structure-based virtual screening

## Abstract

The cannabinoid receptor 1 (CB1R) plays a pivotal role in regulating various physiopathological processes, thus positioning itself as a promising and sought-after therapeutic target. However, the search for specific and effective CB1R ligands has been challenging, prompting the exploration of drug repurposing (DR) strategies. In this study, we present an innovative DR approach that combines computational screening and experimental validation to identify potential Food and Drug Administration (FDA)-approved compounds that can interact with the CB1R. Initially, a large-scale virtual screening was conducted using molecular docking simulations, where a library of FDA-approved drugs was screened against the CB1R’s three-dimensional structures. This in silico analysis allowed us to prioritize compounds based on their binding affinity through two different filters. Subsequently, the shortlisted compounds were subjected to in vitro assays using cellular and biochemical models to validate their interaction with the CB1R and determine their functional impact. Our results reveal FDA-approved compounds that exhibit promising interactions with the CB1R. These findings open up exciting opportunities for DR in various disorders where CB1R signaling is implicated. In conclusion, our integrated computational and experimental approach demonstrates the feasibility of DR for discovering CB1R modulators from existing FDA-approved compounds. By leveraging the wealth of existing pharmacological data, this strategy accelerates the identification of potential therapeutics while reducing development costs and timelines. The findings from this study hold the potential to advance novel treatments for a range of CB1R -associated diseases, presenting a significant step forward in drug discovery research.

## 1. Introduction

Structure-based virtual screening (SBVS) is a computational method for early stage drug discovery starting from novel bioactive molecules [1,2]. Its application appears more efficient than traditional drug discovery approaches, which are often rather complex, expensive and risky.

To be applied, SBVS needs available three-dimensional structures of the proteins of interest, which can be obtained from different experimental techniques such as X-ray diffraction and cryogenic electron microscopy (cryo-EM), and more recently Alphafold—an artificial intelligence that predicts the protein conformation from the amino acid sequence [3]. Then, SBVS can be used for a potent drug discovery technique named drug repurposing (DR), which starts from existing drugs that were commercialized for different therapeutic indications. Of note, DR increases the success rate of drug development, a process that takes more than 10 years and EUR 1 billion per drug, with a high (~90%) failure rate in clinical trials [4].

The combination of SBVS and DR leads to structure-based drug repurposing (SBDR), a technique that allows us to discover potentially new drugs from virtual libraries of approved compounds, by uncovering their interactions with selected proteins. The prediction of drug–protein interactions and the analysis of their binding affinity values can be exploited in different computational methods, such as molecular docking, alchemical binding free energy calculation, umbrella sampling and molecular mechanics/generalized Born and surface area solvation (MM-GBSA) or molecular mechanics/Poisson–Boltzmann surface area (MM-PBSA) [5].

DR also promises high approval rates, because the safety of any commercially available drug has been already assessed in preclinical and clinical trials; thus, the time (and money) needed for drug development can be reduced using this approach [6,7].

Aspirin is the oldest example of DR. Initially formulated by Bayer in 1899 as an analgesic, it underwent repurposing several decades later as an antiplatelet aggregation drug [8,9]. Then, aspirin was proposed to treat other conditions such as cancer [10,11] and cardiovascular diseases [12]. Thalidomide is another example of DR. It was first marketed in 1957 for the treatment of anxiety, sleeping trouble and morning sickness. Yet, it was soon withdrawn because of its teratogenicity [13,14,15], but later on, it was repurposed to treat cancers—particularly erythema nodosum leprosum, multiple myeloma and other hematological malignancies [16]. Maybe the most famous example of DR is sildenafil, that was developed for angina but then repurposed for erectile dysfunction [17]. Finally, among repurposed drugs worth mentioning, one can list baricitinib, remdesivir and tocilizumab, originally developed to treat Alopecia Areata, viral infections (SARS, MERS and AIDS) and rheumatoid arthritis, respectively [18,19,20], but then repurposed as potential COVID-19 treatments [21,22].

Here, we sought to use SBDR for repurposing Food and Drug Administration (FDA)-approved drugs on cannabinoid receptor 1 (CB1R), one of the most prominent G protein-coupled receptors in the human brain [23,24,25]. To this end, knowing the 3D structure details of CB1R is extremely important. This receptor shows the classical 7 transmembrane fold with a lid above the binding site that contains abundant acidic residues and a highly hydrophobic orthosteric binding pocket [26,27]. In addition, a ‘twin toggle switch’ of Phe200 and Trp356 appears essential for CB1R activation [28]. Overall, CB1R is considered a major pharmacological target, due to its many implications for diseases of the central nervous system (CNS) and peripheral organs [24,29]. Unsurprisingly, CB1R is widely distributed throughout the body and is highly expressed within the CNS (approximately 10 to 50 times more than receptors of classic neurotransmitters like opioid and dopamine receptors) in the basal ganglia, hippocampus, cerebellum, amygdala, cingulate cortex, medial hypothalamus and spinal cord [30]. Moreover, CB1R is present—though to a lesser extent—in the periphery, namely in adipose tissue, liver, skeletal muscles, kidney and pancreas [21,31,32,33,34].

CB1R was first discovered as the target of the main psychoactive ingredient of cannabis extracts, Δ^9^-tetrahydrocannabinol [24,35,36]. Later on, it was recognized that CB1R is a key element of a complex lipid signaling system, called the endocannabinoid (eCB) system [24,37]. The latter comprises eCBs, that are endogenous ligands of CB1R [24,37]. The most relevant eCBs derive from arachidonic acid, anandamide (*N*-arachidonoylethanolamine, AEA), which is an amide, and 2-arachidonoylglycerol (2-AG), which is an ester. In addition to CB1R, AEA and 2-AG have other receptor targets and a number of metabolic enzymes that have been recently reviewed in detail [24]. Here, only the main metabolic enzymes of 2-AG (biosynthesis: diacylglycerol lipases (DAGL) α and β; degradation: monoacylglycerol lipase (MAGL) and α/β hydrolase domain-containing (ABHD) proteins 6 and 12) [24,38] have been analyzed, along with fatty acid amide hydrolase (FAAH), the main catabolic enzyme of AEA [24,37].

During the last decade, hundreds of compounds able to bind to CB1R have been synthesized and tested, reporting diverse pharmacological effects [24,39,40,41]. Unfortunately, most of them have been withdrawn because of undesirable side effects. Noteworthy seems the case of SR141716A (SR1, also known as Rimonabant or Acomplia^®^). This selective antagonist/inverse agonist of CB1R was first approved in Europe in 2006 for the management of obesity, but it was then withdrawn two years later because of adverse effects like an increased incidence of depression and suicidal ideation [42,43]. Another potent synthetic CB1R agonist, called AMB-Fubinaca, has been named the “zombie drug” in 2016, because 33 people (25–59 year old) were adversely affected with a semicomatose state [44,45]. These examples simply remind us that there is still an urgent need to find effective drugs able to modulate CB1R with minimal (if at all) side effects.

Here, SBDR has been used on two different 3D structures of CB1R, in order to increase the accuracy of the calculation taking into full account the orthosteric site flexibility of the receptor. Indeed, CB1R shows different conformations upon binding lead by molecular properties. Significant structural modifications due to the ligand nature are evident in helices I and II [28]. In particular, helix I undergoes an inward bending of approximately 6.6 Å, while helix II rotates inwardly by approximately 6.8 Å compared to the agonist-bound and the antagonist-bound states [28]. Likewise, notable conformational changes are observed in the cytoplasmic section of the receptor, where helix VI moves outward by about 8 Å. As a result of the inward shifts of helices I and II, the ligand-binding pocket’s volume reduces from 822 Å^3^ in the antagonist-bound structure to 384 Å^3^ in the agonist-bound complex, signifying a substantial 53% reduction [28,46].

In pursuit of identifying commercial drugs capable of interacting with CB1R, an FDA-approved list of 1379 molecules was retrieved from a chemical database (Zinc15 database). To further validate the in silico results, in vitro competitive radioligand assays were also performed and the experimental binding affinity of the compounds were calculated. Radioligand binding assays are indeed a powerful tool in the early phase of the drug design and discovery process, because they allow us to study directly ligand–receptor interactions [47]. Then, a proteomic technique was used to evaluate the possible interaction of the most potent CB1R ligands towards other key elements of the eCB system, in order to predict possible side effects.

## 2. Results

### 2.1. Virtual Screening

**Figure 1:**
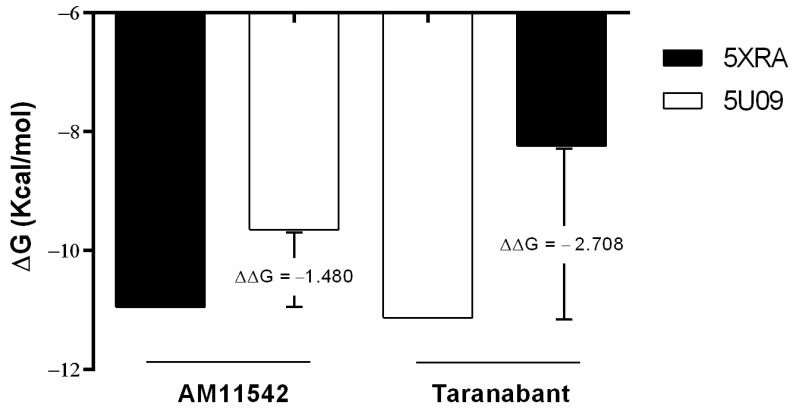
Cross-docking using two 3D structures (PDB 5XRA and 5U09) with AM11542 and Taranabant. ΔΔG = ΔGre-dock − ΔGcross-dock.

To estimate the binding affinity of the investigated drugs towards CB1R, a molecular docking analysis was performed by means of the MOE (Molecular Operating Environment) software (Chemical Computing Group (CCG), Montreal, QC H3A 2R7, Canada). To this end, two different 3D structures of CB1R were used, with PDB codes 5XRA (at 2.80 Å resolution) and 5U09 (at 2.60 Å resolution) in the Protein Data Bank (PDB) (www.rcsb.org). In particular, the first structure is co-crystallized with the receptor agonist AM11542 (Ki = 0.29 nM), whereas the second is complexed with the inverse agonist Taranabant (Ki = 0.13 nM) [27,28]. The performance of the docking procedure was ascertained by redocking the co-crystallized ligands, AM11542 and Taranabant, that yielded ΔG values of −10.951 kcal/mol and −11.134 kcal/mol, respectively (Figure 1). All binding free variations were obtained through the use of Equation (1) computed using MOE.

In this context, it seems important to recall that CB1R’s conformation is affected by the nature of the ligands [26]. Therefore, cross-docking has also been performed by analyzing the binding affinity of AM11542 for 5U09 and Taranabant for 5XRA, that yielded ΔG = −9.6540 kcal/mol and ΔG = −8.2429 kcal/mol, respectively (Figure 1). The different ΔG values of each CB1R ligand in both structures, −10.951 kcal/mol and −9.6540 kcal/mol for AM11542 and −11.134 kcal/mol and −8.2429 kcal/mol for Taranabant, confirmed the different conformation of the agonist-bound state and the inverse agonist-bound state, with a decrease in the binding affinity (ΔG) of −1.480 and −2.708 kcal/mol, respectively (Figure 1).

Then, the ZINC15 database (https://zinc.docking.org/) of 1379 molecules was used [48], in order to perform virtual screening on both 3D structures of CB1R through a rigid-protein docking approach. All details are reported in the Methods section. In this first analysis, a threshold of the binding affinity of −8.5 kcal/mol has been established in order to obtain a restricted set of 200 molecules.

**Figure 2:**
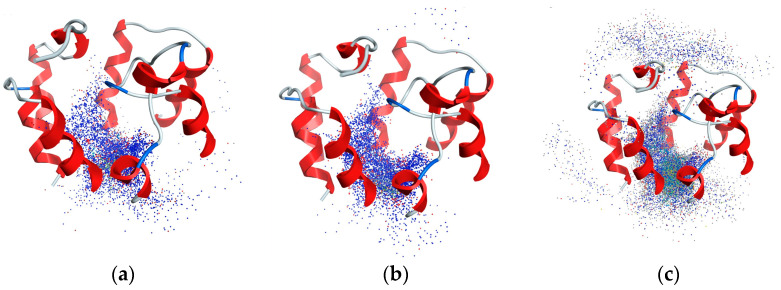
Representative examples of blind docking filter stage showing comparison between AM11542 (**a**), Raloxifene (**b**) and mupirocin (**c**), a discarded drug, in channel of 5XRA structure. Each blue dot represents a predicted pose with high affinity (from −12.0 to −9.0 kcal/mol). Light blue dots are poses with medium affinity (from −8.0 to −7.0 kcal/mol).

Next, to reveal only potential CB1R agonists among the selected molecules, all subsequent analyses were carried out on the 5XRA structure [2]. Afterwards, a blind induced-fit docking, localized in the channel that leads to the orthosteric site of the 5XRA structure, was performed using an induced-fit approach, which allows the binding site to move freely. For this purpose, a second filter stage, that selects only the molecules with several high affinity poses close to the binding site, was applied. Figure 2 shows a representative example of the comparison between AM11542 (a) and Raloxifene (b), with all poses into the channel, and a discarded compound (mupirocin) (c), with several scattered predicted poses.

These channel analyses may provide an explanation for the different potency and selectivity of the CB1R ligands [49,50]. With this filter, 10 drugs were selected: Aminopterin (APGA), Avanafil, Ceftriaxone, Methotrexate, Miltefosine, PGE-1, Raloxifene, Raltegravir, Riociguat and Valsartan, as shown in Table 1. Interestingly, among these compounds, Methotrexate and APGA show similar structures, also shown in Table 1 for comparison.

The selected compounds have different chemical structures, and expectedly, they have been marketed for different diseases such as cancer, hypertension, HIV, leukemia, erectile dysfunction and osteoporosis (Table 1). Of note, one of the selected drugs, Raloxifene, has been already reported to bind to CB1R with Ki = 210 nM [51].

### 2.2. Analysis of CB1R Binding

With the aim of validating in silico computational data, competitive radioligand binding assays were performed at 10 µM to estimate the drug potency. The potent CB1R synthetic cannabinoid [^3^H]CP55,940 was used as the ligand to be displayed in mouse brain membranes. In the same experiments, Raloxifene was used as a positive control [51].

**Figure 3:**
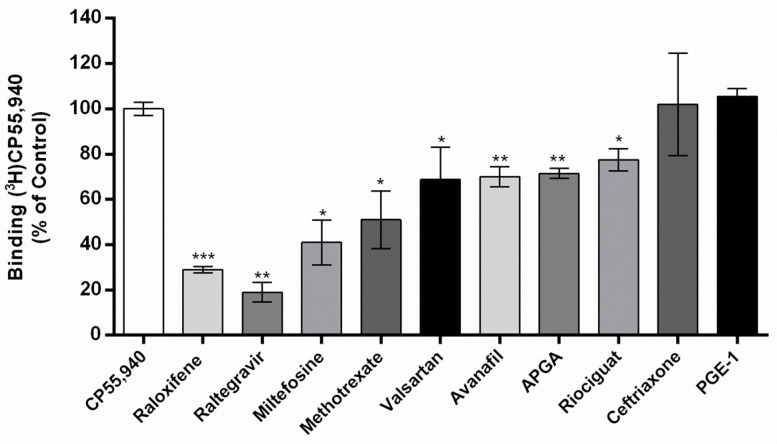
Competition of [^3^H]CP55,940 binding by Aminopterin (APGA), Avanafil, Ceftriaxone, Methotrexate, Miltefosine, PGE-1, Raloxifene, Raltegravir, Riociguat and Valsartan at 10 µM in membrane mouse brain. Data are presented as the mean (±SD) of two independent experiments, each performed in duplicate. * *p* < 0.05, ** *p* < 0.01 and *** *p* < 0.001.

Eight of the ten selected drugs significantly competed with [^3^H]CP55,940 (Figure 3).

In particular, the most potent appeared to be Raltegravir, that produced ~80% displacement of [^3^H]CP55,940. Interesting, Raloxifene was less potent than Raltegravir (Figure 3). Furthermore, Miltefosine and Methotrexate displaced ~60% and ~50% [^3^H]CP55,940, respectively. Instead, Valsartan, Avanafil, APGA and Riociguat were found to be weak competitors, that produced ~30% (the first three) and ~20% displacement of [^3^H]CP55,940, respectively (Figure 3). Finally, Ceftriaxone and PGE-1 failed to show any competition at CB1R (Figure 3), which in the case of PGE-1 could be due to its poor stability [52].

### 2.3. Activity-Based Protein Profiling

**Figure 4:**
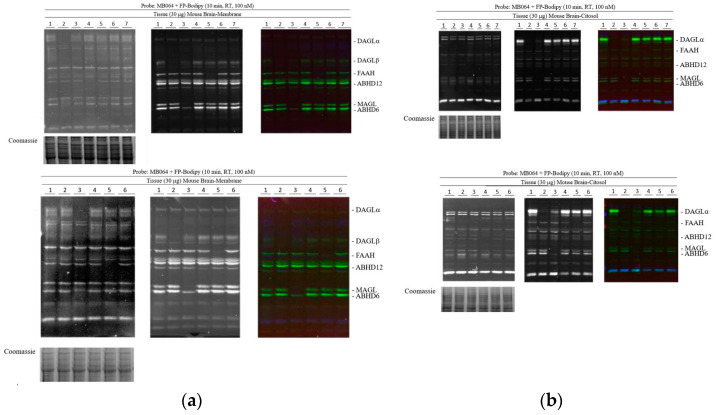
Activity-based protein profiling of some of the main enzymes of the endocannabinoid system in the mouse brain, either in membrane fractions (**a**) or in cytosolic fractions (**b**). The samples are in the following order: control (DMSO), DH376, ABX1431, DO264, PF04458745, Raltegravir and Methotrexate (top panels); control (ethanol), DH376, ABX1431, DO264, PF04458745 and Miltefosine (bottom panels).

Competitive activity-based protein profiling (ABPP) was performed to assess potential interactions of the most potent drugs—Raltegravir, Methotrexate and Miltelfosine—excluding the positive control Raloxifene, with some of the key enzymes involved in eCB metabolism. ABPP is a powerful technique that takes advantage of chemical probes able to react with an amino acid located in the catalytic site of the target enzymes [53,54]. Here, membrane and cytosol fractions of mouse brain were used to analyze the interactions of FDA drugs with the following serine hydrolases: DAGLα/β, MAGL, ABHD6, ABHD12 and FAAH (Figure 4).

To this end, two ABPP probes (MB064 and FP-Bodipy) were used at 100 nM and 10 µM, respectively [55]. Moreover, inhibitors of DAGLα/β (DH376), MAGL and ABHD6 (ABX1431), ABHD12 (DO264) and FAAH (PF04458745) were used as positive controls. None of the tested drugs were able to interact with the selected enzymes, supporting the lack of off-targets (Figure 4).

### 2.4. Cell Viability

**Figure 5:**
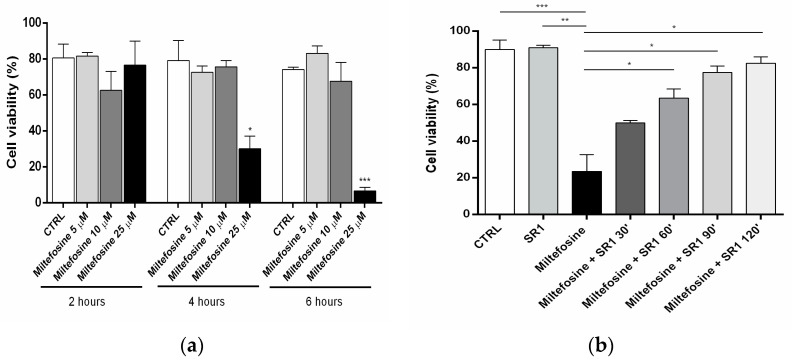
Dose- and time-dependence of HaCaT cell viability upon Miltefosine exposure (**a**). Live cells percentage after 4 h of incubation with Miltefosine (25 µM) and SR141716 (1 µM) at different preincubation times (30 min, 1 h, 90 min and 2 h) (**b**). * *p* < 0.05, ** *p* < 0.01 and *** *p* < 0.001.

The mechanisms of action of FDA-approved drugs used in this study are known [56,57], except for Miltefosine, whose molecular targets have not been identified yet. However, this drug is known to inhibit the proliferation of human keratinocytes (HaCaT cells), at micromolar concentrations [58]. In line with this, and keeping in mind the role of CB1R in cancer [59], here, the anti-proliferative effects of Miltefosine were investigated in the micromolar range (Figure 5a).

Furthermore, the effects were analyzed at different incubation times (2, 4 and 6 h), in order to assess possible time-dependence (Figure 5b). Miltefosine failed to affect cell growth at lower concentrations and at any time of incubation. Instead, at 25 µM, it showed anti-proliferative effects that were time-dependent (Figure 5a,b). Remarkably, the CB1R antagonist SR1 attenuated the effect of Miltefosine, in a manner that was directly proportional to the preincubation time (Figure 5b). These results demonstrate that Miltefosine exerts an anti-proliferative effect via the engagement of CB1R.

## 3. Discussion

DR represents an efficient approach to drug discovery because it exploits approved drugs with a known safety profile. The main advantage of DR is the marked reduction in the costs of clinical trials, which account for ~60% of the total cost of drug development. Indeed, DR can lead to new therapeutic indications for existing drugs with extensive clinical history and toxicology.

Here, virtual screening performed on two different 3D structures of CB1R allowed us to select 200 molecules out of 1379 compounds reported in the ZINC15 database. Then, 10 compounds were isolated through the application of a second filter to find possible agonists for CB1R using the 5XRA structure (Table 1). To validate the screening method employed, one of the final compounds has previously been reported to bind to CB1R. This selective estrogen receptor modulator, Raloxifene, exhibited a Ki value of 210 nM on CB1R [51]. Additionally, the authors investigated various compounds belonging to five structurally distinct classes of selective estrogen receptor modulators [51]. Notably, all the tested molecules demonstrated weaker affinities for the receptor compared to Raloxifene, such as Bazedoxifene, Nafoxidine and Ospemifene. Importantly, the initial filtering stage selected only the most potent compound among the estrogen receptor modulators. Indeed, the chosen threshold of −8.5 kcal/mol discarded Ospemifene with a ΔG of −8.28 kcal/mol, as well as Nafoxidine and Bazedoxifene with a ΔG ~ −8 kcal/mol. Following the computational analysis, an experimental competitive binding assay was conducted to validate the in silico findings. Specifically, eight drugs displayed the ability to displace the potent CB1R agonist, CP55,940, with a moderate or high affinity (Figure 3) and achieving a prediction accuracy of 80%. Among them, Methotrexate, Miltefosine and Raltegravir as well as the positive control, Raloxifene, showed the most significant displacement of [^3^H]CP55,940, exceeding 50% (Figure 3). The doses and characteristics of these compounds are detailed in Table 2.

Furthermore, to explore potential interactions between the selected drugs and representative eCB system enzymes and the related ones, a proteomic analysis of ABHD6/12, DAGL, FAAH and MAGL was performed. The results revealed that the tested compounds did not interact with any of these enzymes (Figure 4). Although the analyzed drugs are highly established in treating specific diseases [56,57], their interaction with CB1R may extend therapeutic actions or elucidate adverse effects through synergistic effects. For instance, the effects of Raloxifene on estrogen receptors and CB1R could account for both beneficial actions and potential adverse events [51]. The binding assay demonstrates the ability of these compounds to interact with CB1R; however, it is essential to consider the dosage for each drug. Raltegravir, the most potent compound, is used in combination with other medications to treat human immunodeficiency virus (HIV) infections (Table 1 and Table 2) [60]. It belongs to the class of medications called HIV integrase inhibitors, and the initial regimen involves 400 mg (0.9 mmol) tablets taken twice daily, with a relatively high concentration (Table 2). Some reported side effects include insomnia, abnormal dreams, loss of appetite, headache, nausea, vomiting, fast heartbeat and depression, which may partially be attributed to CB1R involvement. Methotrexate, on the other hand, belongs to the class of medications called antimetabolites, and its dosage depends on the specific pathology being treated. It is used to slow the growth of cancer cells in certain types of cancer and is also employed to treat severe psoriasis and rheumatoid arthritis by reducing the immune system activity (Table 1 and Table 2). Interestingly, THC has shown similar effects to Methotrexate in neutralizing the inflammatory process [61]. Daily oral administration of THC for 21 days in arthritic rats was well tolerated, without causing significant psychoactive side effects; simultaneously, it attenuated the severity of clinical manifestations [61]. Notably, patients with rheumatoid arthritis and osteoarthritis have high expression levels of CB1R [61]. The interaction between Methotrexate and CB1R highlighted in this study could be involved in the compound’s capacity to treat psoriasis and rheumatoid arthritis.

Among the tested drugs, Miltefosine is particularly interesting as a classic multi-target drug. Originally developed in the 1980s as an antineoplastic agent, it is now marketed for leishmaniasis and has been approved for human treatment since 2002 [62], due to repurposing studies. Another noteworthy feature of Miltefosine is its amphiphilic nature, bearing remarkable similarity to endocannabinoids, hence explaining its ability to bind to CB1R (Table 1). Additionally, Miltefosine has shown to inhibit the proliferation of HaCaT cells [58]. One of the goals achieved in this study lies in establishing a clear relationship between Miltefosine’s ability to bind to CB1R and its effect on HaCaT cells. Notably, this cell line expresses the full eCB system [63]. The data illustrate that the selective antagonist/inverse agonist SR1 inhibits the antiproliferation effect of Miltefosine in a time-dependent manner (Figure 5b), demonstrating that its mechanism of action is mediated by CB1R. Miltefosine is administered orally at a dosage of 50 mg (0.122 mmol) three times a day, with common side effects including decreased appetite, diarrhea and vomiting.

CB1R plays a critical role in several human physiological and pathological conditions. Therefore, extensive endeavors have been undertaken to develop ligands for treating various diseases; nevertheless, none of these ligands are currently utilized in clinical settings.

## 4. Materials and Methods

### 4.1. Materials

Chemicals were of the purest analytical grade (>95%). [^3^H]CP55,940 (126 Ci/mmol) was from Perkin Elmer Life Sciences, Inc. (Boston, MA, USA). Avanafil, Aminopterin, Ceftriaxone, CP55,940, Dofetilide, Methotrexate, Miltefosine, Prostaglandin E1, Raloxifene, Raltegravir, Riociguart and Valsartan were from Cayman Chemical Company, Ann Arbor, MI, USA. Human keratinocytes (HaCaT cells) were provided by CLS Cell Lines Service GmbH, (Eppelheim, Germany).

### 4.2. Virtual Screening

The crystal structure of CB1R, with a potent co-crystallized agonist AM11542 (PDB code: 5XRA), was retrieved from the PDB (www.rcsb.org), and the Experimental Data Snapshot and the PDB validation were analyzed: resolution (2.80 Å), RFree (0.254), Clashscore (6), Ramachandran outliers (0), sidechain outliers (0.6%) and RSRZ outliers (4.7%). The CB1R crystal structure with a potent inverse agonist Taranabant (PDB code: 5U09) was retrieved from the PDB (www.rcsb.org), and the Experimental Data Snapshot and the PDB validation were analyzed: resolution (2.60 Å), RFree (0.247), Clashscore (14), Ramachandran outliers (0), sidechain outliers (0) and RSRZ outliers (0.8%).

#### 4.2.1. Proteins and Ligands Preparation

The simulation studies were carried out using the cutting-edge Molecular Operating Environment (MOE 2021.0102) by the Chemical Computing Group (2021). For the selected crystal structure, the “Structure Preparation” panel with the “Protonate 3D” function was employed to optimize the ionization states of the added hydrogen atoms.

Atoms further away than 8 Å from the co-crystalized ligand were fixed while constraints were applied to atoms within the active site before the final minimization step to refine the protein structure. The 3D structures of the molecules under investigation were sourced from the Zinc Database (https://zinc.docking.org/) [48]. Ligands in ZINC15 were prepared (protonation and tautomers generation at physiological pH) through ChemAxon’s Jchem while Omega was used to obtain the conformations for docking.

#### 4.2.2. Re-Docking Validation

To validate the molecular docking algorithm, co-crystallized ligands (AM11542 and Taranabant) were re-docked into the orthosteric site. The validation was considered successful when the ligand conformation with the lowest energy score closely overlapped with the co-crystallized molecule in the protein X-ray structure, achieving an impressive RMSD < 2.0 Å [64,65].

#### 4.2.3. Protein Rigid Docking

Initially, a protein rigid docking using FDA drugs was performed using 5XRA and 5U09 structures. The dock panel settings included the Triangle Matcher method for placement and the London dG (Equation (1)) as the scoring function [66]. This method takes into account several factors, including rotational and translational entropy (c), energy due to the loss of flexibility of the ligand (*E*_flex_), geometric imperfections of hydrogen bonds (*f*_HB_), geometric imperfections of metal ligations (*f*_M_) and desolvation energy (*D*_i_).

All drugs demonstrating a binding affinity of at least −8.5 kcal/mol were identified in both structures. Subsequently, these molecules underwent further screening through an additional filter stage.

#### 4.2.4. Blind Induced-Fit Docking

A blind docking approach was then conducted, specifically targeting the extracellular domain region up to the orthosteric site within the 5XRA structure. In this phase, an induced-fit docking strategy was employed, allowing for flexibility within the binding site.

An induced-fit refinement was executed, enabling both the ligand and the active site to move freely. The resulting poses were scored using the London dG scoring function.

(1) $$∆G=c+E_{flex}+\sum_{h-bonds}c_{HB}f_{HB}+\sum_{m-lig}c_{M}f_{M}+\sum_{atomsi}{∆D}_{i}$$

**Scheme 1:**
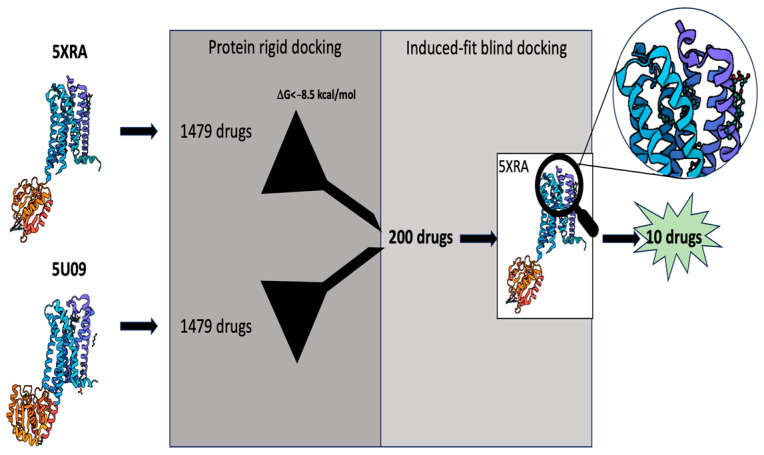
Graphic phases of the virtual screening.

The graphic phases of the virtual screening are shown in the following Scheme 1.

### 4.3. Binding Assay

Mouse brain was resuspended in 2 mM Tris–EDTA, 320 mM sucrose, 5 mM MgCl_2_ (pH 7.4), homogenized in a Potter homogenizer, centrifuged three times at 1000× *g* (10 min) and the pellet was discharged. The supernatant was further centrifuged at 18,000× *g* (30 min), and the resulting pellet was then resuspended in HBSS. For the rapid filtration assays, 50 μg of these membrane fractions were used for each test, along with the radiolabeled agonist [^3^H]CP55,940 at a concentration of 400 pM. The effect of different drugs on CB1R binding was tested by adding the substance, with 80 min of preincubation, directly to the incubation medium followed by incubation time of 40 min at 37 °C [67].

### 4.4. Activity-Based Protein Profiling (ABPP)

Mouse brain lysate (14.6 μL, 2.0 mg/mL lysate, cytosol or membrane fraction) was pre-incubated with vehicle or drugs (0.375 μL, 30 min, 37 °C) followed by an incubation with the activity-based probe (100 nM of MB064 and 100 nM of Bodipy, 10 min, RT). Reactions were quenched with 5 μL Laemmli buffer + β-mercaptoethanol. The reaction was resolved on a 30% acrylamide SDS-PAGE gel (180 V, 75 min). Gels were scanned using Cy 2 (80 s), Cy3 (120 s) and Cy5 (10 s) multichannel settings and subsequently stained with Coomassie after scanning. Fluorescence was normalized to Coomassie staining and quantified with Image Lab (Bio-Rad) [53,54].

### 4.5. Cell Viability

The HaCaT cells are an aneuploid immortalized keratinocyte cell line from adult human skin [68]. HaCaT cells were cultured at 37 °C, 5% CO_2_ in DMEM supplemented with 10% (*v*/*v*) fetal bovine serum (Gibco) and penicillin (100 unit/mL)-streptomycin (0.1 mg/mL). Miltefosine was used at 25 µM, while SR1 was at 1 µM. The incubation time of Miltefosine count was 30 min, and after a further 4 h, the percentage of viable cells was assessed. SR1 underwent a preincubation period of 30 min, 1 h, 1 h and 30 min, and 2 h, allowing for a comprehensive evaluation of its effects.

## 5. Conclusions

This study presents a compelling demonstration of how a combination of computational and experimental approaches can shed light on CB1R as a promising new target for marketed drugs. Moreover, the quest to design selective and safe drugs for CB1R can be addressed through a novel strategy. To this aim, compounds that act on specific targets and only partially interact with CB1R offer a potentially safer therapeutic approach, minimizing adverse effects compared to direct CB1R-targeting molecules. Raloxifene, Raltegravir and Miltefosine could represent an example of this strategy. Considering the widespread distribution of CB1R and its involvement in diverse signaling cascades, further investigations similar to ours become imperative to evaluate its role in the mechanisms of certain drugs.

## Figures and Tables

**Table 1:** Chemical structures and applications of drugs obtained from computational screening.

Drugs	Chemical Structures	Indications
Aminopterin (APGA)	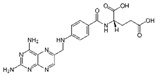	Pediatric leukemia
Avanafil	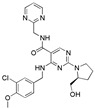	Erectile dysfunction
Ceftriaxone	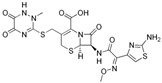	Bacterial infections, such as endocarditis, meningitis, pneumonia, skin infections, urinary tract infections
Methotrexate	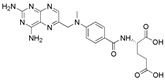	Cancer, Autoimmune diseases, Ectopic pregnancies
Miltefosine	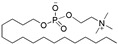	Leishmaniasis, Breast cancer (topical treatment)
PGE-1	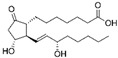	Erectile dysfunction, Neonatal congenital heart defects
Raloxifene	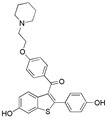	Osteoporosis, Breast cancer prevention
Raltegravir	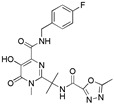	HIV
Riociguat	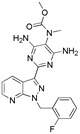	Pulmonary hypertension
Valsartan	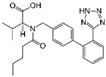	Hypertension, Heart failure, Diabetes, Kidney disease

**Table 2:** Commercial name, doses and features of drugs with ability to bind to CB1R.

Drugs	Commercial Name	Dose	Features
Methotrexate	Trexall, Otrexup, Rasuvo, Xatmep, RediTrex, Jylamvo	Dosage depends on pathology	Methotrexate is a folate derivative that inhibits several enzymes responsible for nucleotide synthesis. It is used to treat inflammation caused by arthritis or to control cell division in neoplastic diseases.
Miltefosine	Impavido	50 mg BID/TID	Miltefosine is a broad spectrum antimicrobial, anti-leishmanial, phospholipid drug developed in the 1980s as an anti-cancer agent.
Raltegravir	Isentress	600 mg BID	Raltegravir is an antiretroviral agent used for the treatment of HIV infections. It is the first of a new class of HIV drugs.

Notes: BID, bis in die; TID, ter in die.

## Data Availability

Data are contained within the article.
